# Innovative Coatings Based on Peppermint Essential Oil on Titanium and Steel Substrates: Chemical and Mechanical Protection Ability

**DOI:** 10.3390/ma13030516

**Published:** 2020-01-22

**Authors:** Martina Cazzola, Sara Ferraris, Giuliana Banche, Giovanna Gautier Di Confiengo, Francesco Geobaldo, Chiara Novara, Silvia Spriano

**Affiliations:** 1 Department of Applied Science and Technology, Politecnico di Torino, 10129 Torino, Italy; martina.cazzola@polito.it (M.C.); sara.ferraris@polito.it (S.F.); Francesco.geobaldo@polito.it (F.G.); chiara.novara@polito.it (C.N.); 2 Centro Interdipartimentale Polito BioMEDLab, Politecnico di Torino, 10138 Torino, Italy; 3 Department of Public Health and Paediatrics, Microbiology Division, University of Turin, 10125 Torino, Italy; giuliana.banche@unito.it; 4 IMAMOTER-National Council of Research, 10135 Torino, Italy; g.gautier@imamoter.cnr.it

**Keywords:** essential oils, natural lubricants, natural coatings, adhesion, stability, scratch, FTIR, protection

## Abstract

A coating that was made of peppermint essential oil was obtained on different metal substrates: Ti6Al4V alloy (mechanically polished and chemically etched) and 316L stainless steel (mechanically polished and mechanically ground). The final aim is to get a multifunctional (chemical and mechanical) protection of metal surfaces in contact with water media. The coatings were characterized by means of fluorescence microscopy, contact angle measurements, and Fourier Transformed Infrared spectroscopy (FTIR) spectroscopy. The chemical stability of the coatings was tested by means of soaking in water for different times (up to seven days) and washing with different alkaline or acidic solutions. The mechanical adhesion of the coating was tested by tape adhesion test (before and after soaking) and scratch tests to verify whether it has protection ability with respect to the metal substrate. All of the performed characterizations show that the coatings are chemically stable on all of the substrates and are nor dissolved or removed by water during soaking or by alkaline solutions during washing. The adhesion is high and classified as 4B or 5B (on the chemically etched or mechanically ground substrates) according to ASTM D3359-97, depending on the substrate roughness, both before and after soaking. In the case of scratch test (up to 10 N), the coating is not removed and it has a protection action that is able to avoid the surface damage, even if the substrate has a plastic deformation.

## 1. Introduction

Traditionally, lubrication and protection of metals is devoted to synthetic fluids, coatings, and paints. Natural (fatty and essential) oils can be considered as an environmentally friendly alternative, because they are biodegradable, not explosive, non-toxic (they do not irritate the skin), renewable, low cost, and easily available from organic waste [1]. The essential oils, such as the peppermint oil used in this research, contain terpenes and terpenoids, and, even if they were less explored than the vegetable ones because of the higher costs, are gaining increasing interest for the realization of corrosion inhibitors and green polymers [2], even if their application in this field is still poorly explored.

Vegetable fatty oils, such as monounsaturated oils (mainly ester-based), are in use as biodegradable lubricants in applications that include engine oils, hydraulic fluids, and transmission oils [3]. They have low volatility, excellent lubricity, higher flash point, refrigerant, and solubilizing capacity for contaminants and additives than mineral base oils. However, extensive vegetable oil use is restricted, owing to the poor fluid flow behavior, solidification at low temperatures, and low oxidative and hydrolytic stability [3]. The most serious problem is their poor oxidative stability, primarily owing to the presence of bis allylic protons. Some vegetable oils, such as linseed oil and rapeseed oil, have a high degree of unsaturation, depending on the amount of linoleic and linolenic acid derivatives. As a result, the thermo-oxidative stability of these oils is poor, resulting in gummy and resinous products at elevated temperatures. Their use as lubricants without a reduction of unsaturation can cause deposit formation, corrosive action, and damage with relatively short useful service life. One of the several ways to reduce unsaturation is to partially hydrogenate these vegetable oils to improve their service lives without affecting the freezing points to a large extent. For some high temperature applications, lauric oils, such as coconut or palm kernel, may be used for optimal stability. Essential oils are less explored as lubricants than the fatty vegetable ones, but some of them (kernel peach oil, grape seed oil, pine oil, carrot seed oil, chamomile oil, laurel oil, eucalyptus oil, lavender oil, and rosemary oil) have been considered as lubricating additives for low sulfur diesel fuels [4]. Better lubricant ability was obtained in the case of the oils rich in polar compounds (kernel peach oil, grape seed oil, camomile oil, laurel oil, and carrot seed oil), with higher density and viscosity.

Moving from lubricant to metals protection, the use of natural oils as corrosion inhibitors, as additives into the liquid medium to reduce its corrosion action, has been reported for stainless steel, aluminum, copper, and zinc alloys [5,6,7,8,9], with an exponential trend of published papers in the last five years, but it is still an open issue and a systematic scientific approach while taking that the composition of the mixtures of different natural compounds is still missing into account. Essential oils and extracts from leaves and seeds of several medicinal plants have been largely investigated as anticorrosion agents against alkaline and acid media, as well as chloride ions [3,4,5,6,7]. The anticorrosion activity is attributed to the presence of heterocyclic constituents, such as alkaloids, flavonoids, tannins, cellulose, and others.

Regarding coatings and paints, the use of natural oils as protective and/or auto-lubricant coatings on metal substrates has been already described in the literature and applied in commercial products [10,11], but its significance and application in the world of fully environmentally friendly coatings can be widely exploited. Drying oils (iodine number higher than 130) form films in their virgin forms, mainly through their unsatured portion, and can crosslink through reaction with oxygen: they dry to a solid and elastic film when exposed for certain periods of time to air. On the other side, non-drying oils can be used as reactive solvents or for free flowing coatings materials, eventually after incorporation of suitable functional groups or modifiers. Essential oils are known to be prone to auto-polymerization and this is usually considered to be detrimental for their stability [12]. The mechanism of polymerization was investigated in details in the case of single compounds (terpenes like pinene and limonene or terpenoids, like carvone and menthol) [2], while it is not completely known in the case of complex mixtures of compounds, such as in natural extracts. In the case of the peppermint oil, a coating that is rich in oxygenated species (menthol, hydroxylmenthofuran, menthyl-acetate) and a minority fraction of β-cubebene is formed, even if the liquid oil has a prevalence of terpenes; menthone is absent from the coating, even if it is one of the main components of the liquid oil, because it does not polymerize if not chemically modified [13].

According to what above reported, it appears that alternatives to both mineral and natural fatty oils are currently needed and that essential oils are of interest to be explored in this context.

In previous works [13,14], the authors investigated a coating that was made of Peppermint essential oil (EO) on a chemically-treated titanium alloy and defined a protocol for the characterization of it; the aim of this previous research was mainly to confer antibacterial properties to metals for biomedical implant applications. Peppermint essential oil is widely known for its flavoring and refreshing properties, as well as for its antibacterial action. Moreover, it can be obtained from plants (Menta Piperita) that are abundant in specific geographical areas (e.g., Piedmont) supporting the local economy. The purpose of the present work is to investigate whether the coating can be obtained on different metal substrates (untreated titanium alloys or stainless steel) and if it is chemically (water soaking, aggressive washings) and mechanically (scratch and peel) stable. This type of coating could have several applications in general purposes objects, laboratory instruments, or industrial mechanical applications as a multifunctional (biological, chemical, and mechanical) protection for metals coupling film forming ability and stability of the formed coating to anti-adhesive properties, with respect to micro-organisms (such as bacteria and fungi).

## 2. Materials and Methods

The following materials were used as substrates for the coating: Ti6Al4V alloy and AISI316L stainless steel mechanically polished (Ti64-MP and AISI316L-MP), Ti6Al4V alloy chemically treated (Ti64-CT), and stainless steel mechanically ground (AISI316L-MG).

Regarding Ti64-MP (ASTM B348, Gr5, Titanium Consulting and Trading, bar 10 mm in diameter) and AISI316L-MP (AISI316LVM–ASTMF138-InTrauma, bar 14 mm in diameter), discs were cut from the bars and polished with SiC papers (up to 2500 grit). In the case of AISI316L-MG, the AISI316 steel discs were ground with SiC papers up to 400 grit.

With regards to Ti64-CT, the same material as for Ti64-MP was used; the discs were polished up to 2500 grit and, in order to increase the coating adhesion through chemical interaction and/or mechanical interlocking, subjected to a patented chemical treatment that consists in acid etching in diluted hydrofluoric acid and controlled re-oxidation in hydrogen peroxide, as described in [15,16].

The substrates were coated with the peppermint Essential Oil (EO-Mentha piperita-EssenzialMenta, Pancalieri, Italy), as described in [13]. A drop of the EO was spread on the surface of the substrate and then polymerized at 37 °C for 48 h. After that time, the samples were washed in ultrapure water and then left drying at room temperature. The coated as-prepared samples will be called: Ti64-MP-C, Ti64-CT-C, AISI316L-MP-C, and AISI316L-MG-C.

The as-prepared coated samples were characterized by means of fluorescent microscopy observations by using a Zeiss LSM900 confocal microscope (Zeiss, Oberkochen, Germany) that was equipped with a fluorescent light source and Scanning Electron Microscopy (SEM, JCM 6000 plus, JEOL, Tokyo, Japan).

A coated sample of mechanically ground steel (AISI316L-MG-C), together with a mechanically ground steel substrate (AISI316L-MG) for control, were tested towards scratch resistance by using a Revetest Scratch Tester (CSM, Revetest machine), which was equipped with a Rockwell C diamond stylus 200 μm in radius that produces a line scratch (4.9 mm long) under a normal load continuously increasing from 1 N to 10 N. The loading rate was 50.04 N/min. with a speed of 27.3 mm/min. Three scratches were generated on the surface using loading rate of 50 N/min. The scratch test parameters were selected according to the ones reported in the literature for the thin polymeric films [17]. Normal load, friction force, and acoustic emission were recorded during the tests and optical pictures of the scratch track were registered at the critical loads. On the basis of the obtained results optical observations of the scratch tracks were considered the most significant data for the surface characterization and, consequently, are the only one reported in the results section. The sample used for the scratch test was then observed at the confocal microscope that was equipped with a fluorescent light source (Zeiss LSM900, Zeiss, Oberkochen, Germany) to investigate coating permanence in the scratch track.

Some coated samples were soaked in 25 mL of ultrapure water in flasks that were covered with an aluminum foil for protection against light radiation in an incubator at 37 °C for different times: 3 or 7 hours (3 h, 7 h), 3 or 7 days (3 d, 7 d). These samples will be called Ti64-MP-C-(3 h or 7 h or 3 d or 7 d, 3 h/7 h/3 d/7 d), Ti64-CT-C-(3 h or 7 h or 3 d or 7 d), AISI316L-MP-C-(3 h or 7 h or 3 d or 7 d).

Some steel coated samples were tested with respect to resistance to typical cleaning solutions; a standard cleaning protocol was used, as reported in [18,19], consisting in cleaning with a wet sponge using an acid solution of H_2_SO_4_ 0.05 M (0.1 N), a basic solution of NaOH 0.1 M (0.1 N) and a commercial degreaser (Vegetale, Nuncas, Milan, Italy). The coated and washed samples will be called AISI316L-MP-C-(H_2_SO_4_ or NaOH or deg).

Fourier Transformed Infrared spectroscopy (FTIR), contact angle measurement, tape test, and fluorescence microscopy observations characterized the as-prepared coated samples, as well as the coated samples after soaking in water or washing with different solutions.

The FTIR analysis in reflectance mode was performed in duplicate between 400 and 4000 cm^−1^ while using a FTIR (Tensor 37 micro-FTIR with Hyperion 2000 Microscope-Bruker Optics, Ettlingen, Germany).

Contact angle measurements were performed with sessile drop method in a microscope (DSA 100, Kruss, Hamburg, Germany) by a drop of 5 µL of ultrapure water deposited with a micropipette on the sample. Ultrapure water was used as wetting fluid to investigate the behavior of coatings in contact with water-based media (which are the most common ones in the majority of applications). The images were acquired with the camera and elaborated by the instrument software (Drop Shape Analysis, Kruss, Hamburg, Germany, with the sessile drop fitting method) for obtaining the value of the contact angle. The measures were performed in triplicate on each kind of sample produced.

The tape test was performed in accordance with the standard ASTM D3359-97 [20] and the coating adhesion after the test was evaluated by optical microscope observations.

Table 1 reports a summary of samples names, features, and characterizations.

## 3. Results

### 3.1. Coating Preparation and Visual Appearance

The synthesis of a coating of peppermint essential oil previously described by the authors [13] is applied here to several metal substrates: Ti6Al4V alloy and AISI316L stainless steel mechanically polished (Ti64-MP and AISI316L-MP), Ti6Al4V alloy chemically treated (Ti64-CT), and AISI316L mechanically ground (AISI316L-MG) with a rough surface finishing. The Ti64-CT substrate is featured by a multiscale roughness, both at the micro and nano scale and by a surface chemistry characterized by the presence of a surface oxide layer with a high density of OH groups, as already described by the authors [15]; the macroscopical appearance of the Ti64-CT surface is of a green-violet color tone, instead of metal grey as the other substrates, because the surface titanium oxide layer is partially transparent in the visible range. The chemical stability and mechanical adhesion of the coatings, respectively, to soaking in water and scratches or peeling, is tested here.

### 3.2. Fluorescence Microscopy

**Figure 1:**
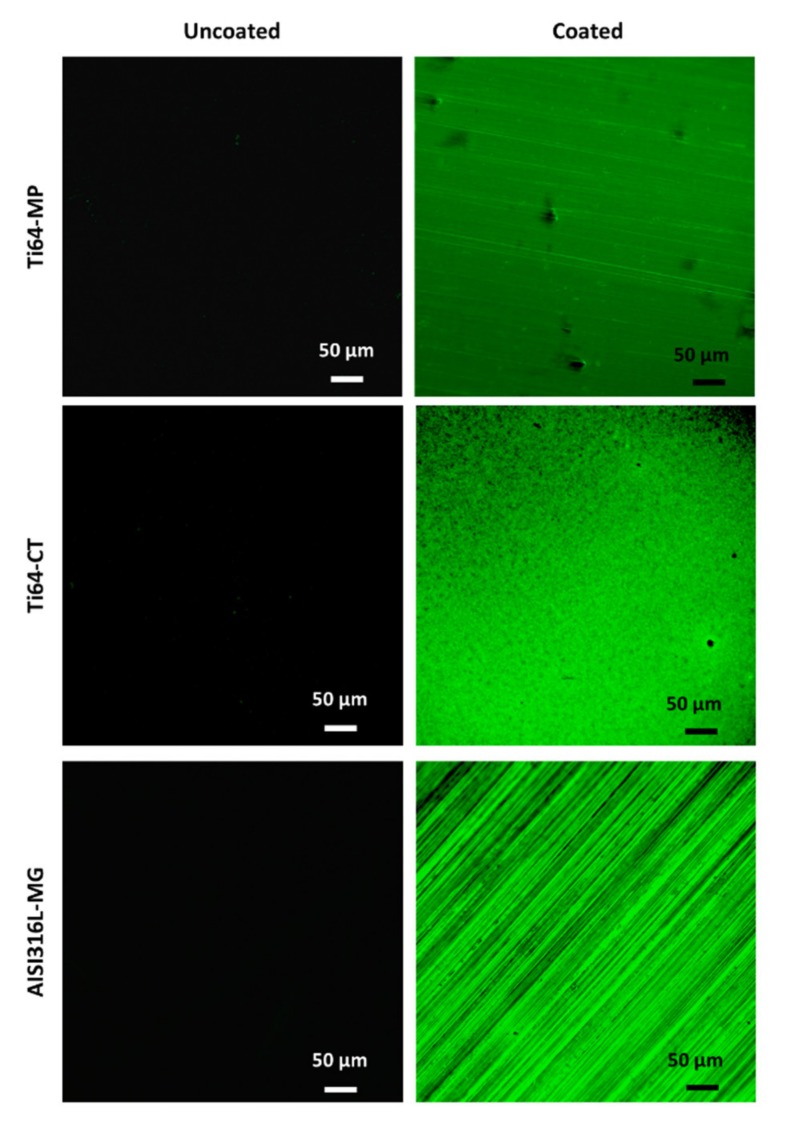
Fluorescent microscopy observations of the coatings obtained: the un-coated substrates are reported on the left (Ti64-MP; Ti64-CT; AISI316L-MG) and the coated surfaces are reported on the right (Ti64-MP-C; Ti64-CT-C; AISI316L-MG-C).

After the procedure of coating, a continuous layer is well visible on all of the tested substrates both by macroscopic observation, as a smooth, transparent and glossy surface, and fluorescence microscopy (Figure 1); in the case of AISI316L, the image of the mechanically ground sample is reported (AISI316L-MG) as an example of the appearance of the coating on this substrate, which is the same for AISI316L-MP-C.

**Figure 2:**
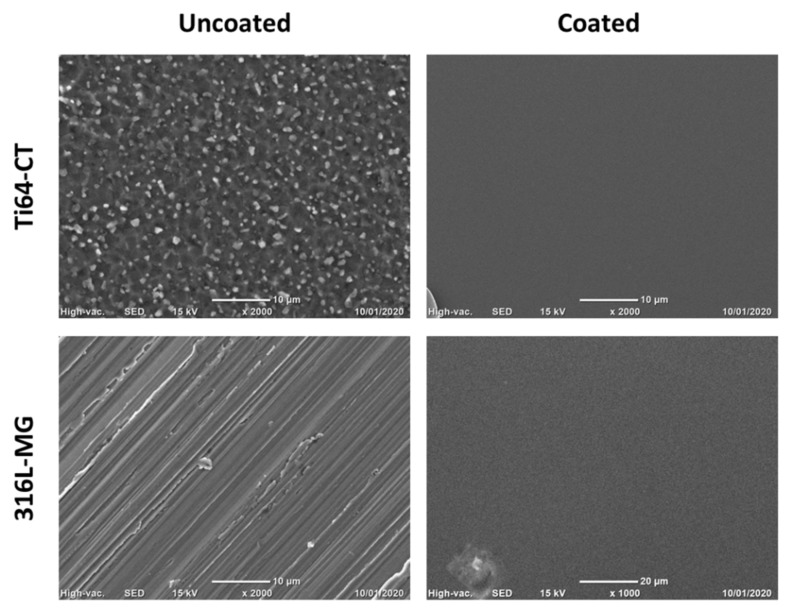
Scanning Electron Microscopy (SEM) observation of Ti64-CT and 316L-MG uncoated and coated samples.

The SEM observations (Figure 2) confirm that the coating is smooth and homogeneous and completely cover the metallic surfaces hiding their topography.

### 3.3. FTIR Spectroscopy

**Figure 3:**
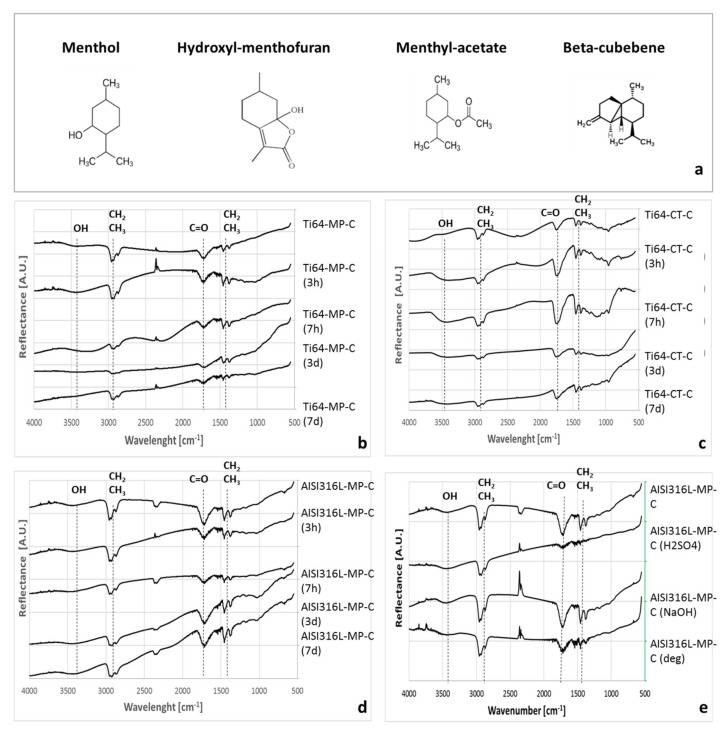
(**a**) Chemical structure of the compounds of the coating; (**b**) Fourier Transformed Infrared spectroscopy (FTIR) analysis of the Ti6Al4V polished and coated samples (Ti64-MP-C); (**c**) FTIR analysis of the Ti6Al4V chemical treated and coated samples (Ti64-CT-C); (**d**) FTIR analysis of the 316L stainless steel polished and coated samples (316L-MP-C). All of the the samples were tested before and after soaking in water for different times (3 h/7 h/3 d/7 d) or, in the case of 316L-MP-C, also after washing by different solutions (**e**). The bands around 2350 cm^−1^ are due to atmospheric CO_2_.

FTIR analysis characterized the coatings (Figure 3). As previously described, the coating contains different oxygenated and not oxygenated monoterpenes: menthol, menthyl-acetate, hydroxyl-menthofuran, and β-cubebene [13] (Figure 3a). Menthol and menthyl-acetate are among the components of the peppermint EO used as source, while hydroxyl-menthofuran and β-cubebene are, respectively, a metabolite of menthofuran and an isomer of α-copaene, which are among the components of the source peppermint EO. The characteristic chemical groups of these compounds mainly include C-Hx and C=O, whose stretching (bands around 2800–3000 cm^−1^ and 1710 cm^−1^ for the two groups, respectively) and bending (bands in the 1450–1370 cm^−1^ range for C-H) vibrations can be detected on all of the as-prepared coated samples, as evidenced in Figure 3. As reference, the substrates without coating were also measured by FTIR and, as expected, no peak related to functional groups was detected (data not shown) on Ti64.MP and AISI316L-MP; Ti64-CT only shows a broad signal in the region of OH groups (around 3400 cm^−1^) due to the high hydroxylation degree of the surface oxide layer formed during the chemical treatment.

**Figure 4:**
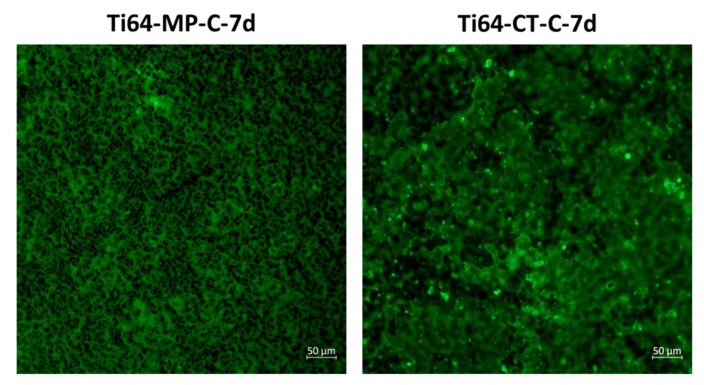
Fluorescent microscopy observations of Ti64-MP-C-7 d and Ti64-CT-C-7 d.

Some of the samples of the different substrates were soaked in water for different times to test the chemical stability of the coatings. After soaking, the coating is still well observable on all samples, even if a slight change in color (from transparent to white) can be observed. The presence of the coating on the samples after soaking was confirmed by FTIR for different soaking times (3 h/7 h/3 d/7 d), as reported in Figure 3 and fluorescent microscopy ([14] and some examples in Figure 4). All of the characteristic bands of the functional group of the oil components are well evident on all of the samples after soaking; a small variability can be observed only in the case of the broad peak of the OH groups. Fluorescent microscopy observations confirm the permanence of the coating after 7 d soaking in water, only a certain porosity can be observed on the surface of the coated samples after soaking, which can be the cause of the observed change in the visual appearance (whitening).

In the case of AISI316L-MP, the stability of the coating was also verified by FTIR after washing with different solutions (an acidic or alkaline diluted solution and a commercial alkaline degreaser). In this case, the spectra of the samples that are washed in an alkaline environment (diluted NaOH or commercial degreaser) are characterized by the same typical coating vibrational bands, while a consistent decrease in intensity of the peaks of the coating is observable after washing in a diluted acid solution.

### 3.4. Contact Angle Measurements

**Figure 5:**
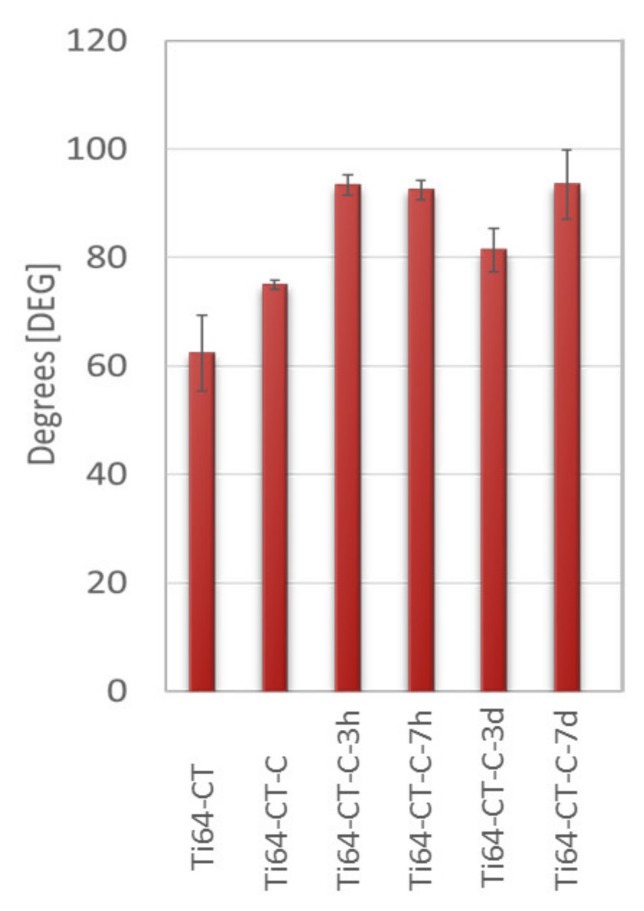
The contact angle values of Ti6Al4V treated substrate before (Ti64-CT) and after coating (Ti64-CT-C), as well as after soaking in water for different times (3 h/7 h/3 d/7 d).

The contact angle measurements were performed on the substrates, the as-prepared coated samples, and the coated samples soaked for different times (3 h/7 h/3 d/7 d). Figure 5 reports the results that were obtained on Ti64-CT. The Ti64-CT substrate is hydrophilic according to the high density of OH groups already described by the authors [15]. A significant increase of the contact angle values and hydrophobic behavior can be detected after the coating formation on this substrate according to the apolar nature of the compounds of the coating. The contact angle values do not change after soaking of the coating in water for different times. On the other substrates, the measurement of the contact angle values is not so significant, because stainless steel 316L and un-treated Ti6Al4V substrates show high contact values, even before the coating formation and the substrate is almost as hydrophobic, as the coating is: that is why no significant change in the contact angle values was detected after the coating formation on these substrates.

### 3.5. Tape Adhesion Test

**Figure 6:**
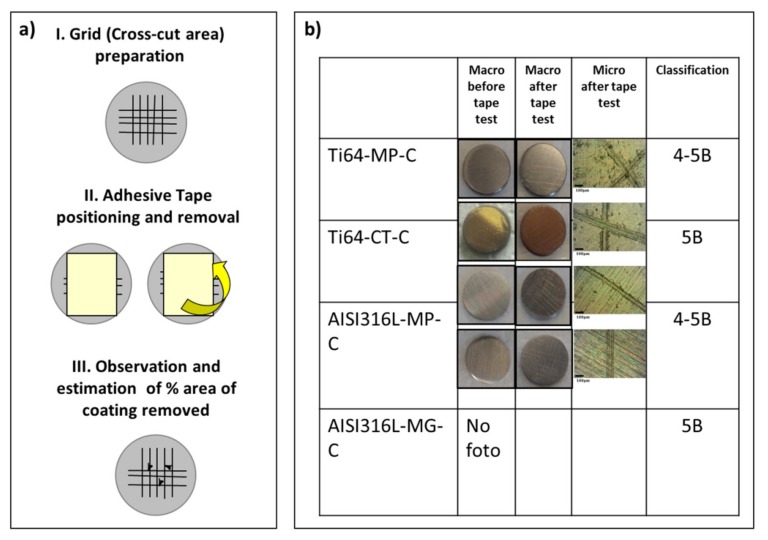
(**a**) Scheme of the tape test, (**b**) Images of the as-prepared coatings (Ti64-MP-C; Ti64-CT-C; AISI316L-MP-C; AISI316L-MG-C) before and after the tape test and classification of coating adhesion according to ASTM D3359-97 standard (5B 0%, 4B < 5%, 3B 5–15%, 2B 15–35%, 1B 35–65%, and 0B > 65% area removed).

The adhesion of the coating to the different substrates was evaluated by tape test, both before and after soaking in water for different times or washing by different solutions. Figure 6a represents a scheme of the tape test performed. Figure 6b reports the images of the as-prepared coatings on the different substrates before and after the tape test; in this case, also a stainless steel substrate, mechanically ground to be more rough before coating (316L-MG), was tested. A halo is observable on the tape after its removal from all of the samples, but the coating is still well observable on all of the samples after the tape test: a peeling limited to a thin outermost layer occurs on all the samples. The white appearance of the samples after soaking for different times is no more observable after the tape test, confirming the peeling of a thin outermost layer. While considering that this removal does not compromise the interface of the coating with the substrate and it has a negligible thickness, it was considered to be negligible. No relevant difference among the samples can be noted. Some small damages and detachment of the coating are observable near the edges of the grid of the Ti64-MP and AISI316L-MP samples. The coatings on the Ti64-CT and SISI316L-MG substrates are less damaged on the edges of the grid than all of the other substrates revealing a higher adhesion of the coating on surface with a higher roughness. Adhesion can be classified as 4-5B for all of the Ti64-MP and AISI316L-MP samples, while it is classified as 5B on the Ti64-CT and AISI316-MG substrates.

**Figure 7:**
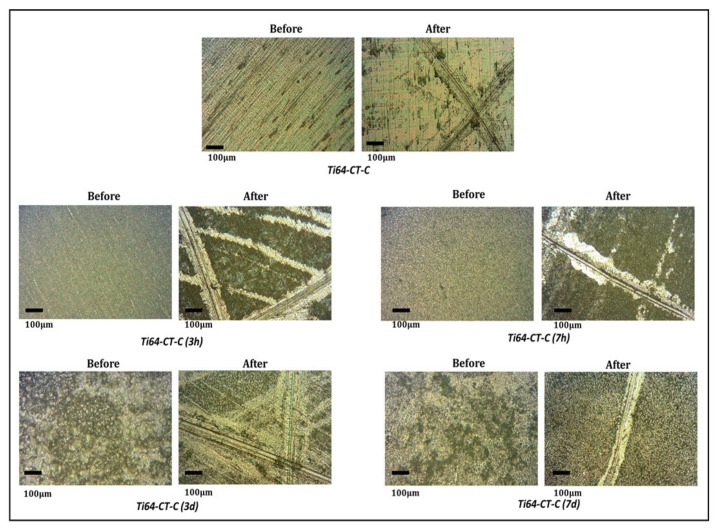
Coatings after soaking for different times (Ti64-CT-C soaked for 3 h/7 h/3 d/7 d) before and after the tape test.

Figure 7 reports the images of the samples soaked for different times, before and after the tape test; the Ti64-CT-C is reported as an example (Ti64-CT-C soaked for 3 h/7 h/3 d/7 d) and the results on the other substrates are analogous. No evident change in the adhesion of the coating due to soaking in water can be observed.

**Figure 8:**
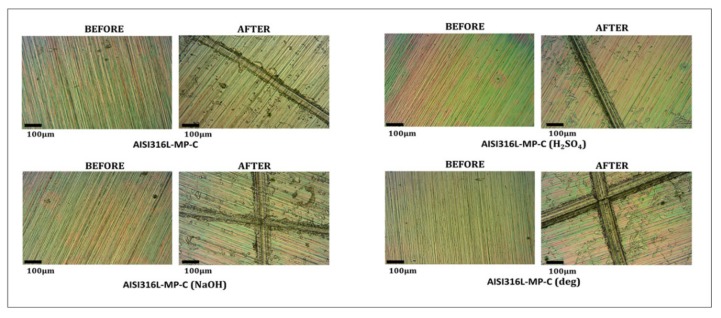
Images of the coating (AISI316L-MP-C) washed with different solutions (NaOH, H_2_SO_4_ diluted solutions or commercial degreaser) before and after the tape test.

Figure 8 reports the images of the samples washed with different solutions, before and after the tape test (AISI316L-MP-C washed with a NaOH, H_2_SO_4_ diluted solutions or commercial degreaser). No evident change in the adhesion of the coating due to washing with different solutions can be observed.

### 3.6. Scratch Test

**Figure 9:**
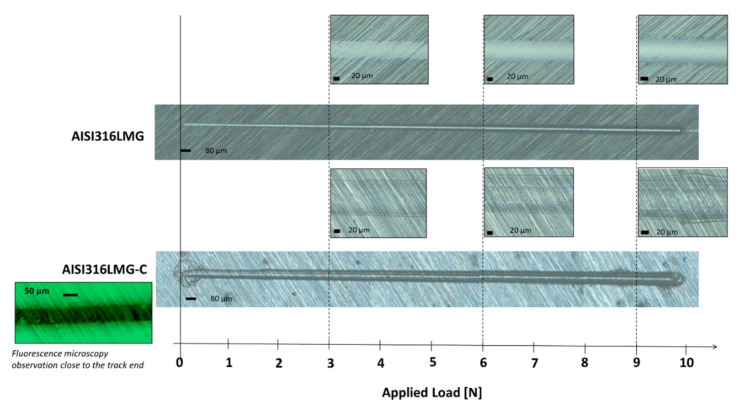
Scratch lines obtained on AISI316L-MP and AISI316L-MP-C.

The samples AISI316L-MG and AISI316L-MG-C were also tested by scratch test and Figure 9 reports the results. On AISI316-MG, it is clear that the scratch induces a plastic deformation of the substrate and a surface damage: a scratch line is well observable and the grooves due to the grinding procedure are no more visible inside the scratch line since almost the beginning of the scratch (see the pictures in correspondence of 3-6-9 N). On the other side, in the case of AISI316L-MG-C, the surface is not damaged (the grooves due to the grinding procedure are well observable in all the pictures of the scratch line) and the coating is not removed from the surface until the maximum load of the test (10 N), even if the scratch line is observable and plastic deformation of the substrate occurs. An image obtained by fluorescent microscopy is also reported as a confirmation of the presence of the coating across the scratch line: even if the deformed material inside the scratch is not at the same focal distance as the coating outside the scratch line, the presence of the coating spread on the scratch track can be appreciated.

## 4. Discussion

As mentioned in the introduction, the investigation of natural oils as lubricants, corrosion inhibitors (liquid additive), and protective coatings for metals is a topic of great scientific interest in the last years. The literature shows an increasing number of published papers and commercial products are on the market. Specifically concerning essential oils, the much more explored topic is their use as liquid corrosion inhibitors, while they are poorly explored as film forming and coatings [9]. It can be seen that often the films formed from virgin vegetable oils do not meet the desirable physico-mechanical and protection requirements while focusing on the specific literature of vegetable oils as paints and coatings [21]. This involves the need of complex chemical processing of the oils and their use only as binders for coatings containing different no environmentally friendly mineral compounds: today, emphasis is on the introduction of paints and coating based on vegetable oils with novel properties, improved performances, fully environment friendliness, and reduced processing steps. That is why the exploration of different types of oils with film forming ability is of interest.

The authors previously investigated the film forming ability of the peppermint essential oil [13] following the above described rationale and the maximization of the local natural sources exploitation. It was assessed that it gives a coating that contains compounds with different chemical functionalities (menthol, menthyl-acetate, hydroxyl-menthofuran, and β-cubebene). The presence of unsaturated carbon bonds is required for polymerization, while the polar groups are adhesion and lubricant promoters. According to this context, the authors explore, in this research, the use of the virgin peppermint essential oil as a drying oil to verify its film forming ability on different substrates, as well as the chemical stability and mechanical adhesion of the formed coating.

The present research shows that a continuous coating is formed both on titanium and steel substrates, as well as both on polished and rough surfaces (on the micro or nano scale) and fluorescent microscopy can be efficiently used to check for the film formation. FTIR measurements on the as-prepared coatings confirm that its chemical composition is well reproducible and it is the same, regardless of the different substrate.

A first requirement in the formation of protective coatings is the creation of a hydrophobic film, which might provide resistance in corrosive media due to reduced wetting by water-based corrosive liquids [22]. Contact angle measurements confirm the hydrophobic behavior of the peppermint oil coating and reveal that it is maintained after soaking for long time in water.

FTIR measurements on the coatings after soaking in water for a long time reveal that the peppermint oil coating is chemically stable and its chemical functionalities are preserved. The FTIR measurements on the samples that were washed in different media reveal the resistance of the coating to the alkaline solutions that is sometimes missing in the coating derived from vegetable oils [21]. In the case of the coating from the peppermint essential oil, the resistance seems to be a bit lower in the case of acid solutions with a thinning of the coating after washing.

The tape and scratch tests both reveal a good adhesion of the coating to the different substrates with an increment, as expected, with the presence of roughness on the substrates at the micro or nano scale. The adhesion is preserved, even after soaking for long time in water and washing in different media.

The scratch test also reveals a good mechanical protection ability of the surface by the coating: the steel surface is preserved from damage, even if the substrate is plastically deformed by a load and the coating is not disrupted, but it is spread across the scratch line.

No significant difference in the coating formation and behavior on the different substrates employed was detected, except that both micro (such as AISI316L-MG) or nano (such as Ti64-CT) rough substrates allow for a better adhesion than the MP ones.

## 5. Conclusions

The possibility to obtain on Ti6Al4V and AISI316L stainless steel a continuous, hydrophobic, well adherent, and chemically stable coating made of a virgin peppermint essential oil is shown here. The coating is fully environmentally friendly and biodegradable. This preliminary investigation can drive a future investigation on the auto-lubricant and anti-corrosion properties of coatings based on essential oils for a multifunctional (chemical and mechanical) protection of metal surfaces that are in contact with water media, where low adhesion of micro-organisms (such as bacteria and fungi) is also required.

## Figures and Tables

**Table 1:** Summary of samples preparation routes, main features, and characterization techniques.

Sample Name	Material	Surface Finishing	Coating	Ageing	Characterizations
Ti64-MP	Ti6Al4V	Mechanical Polishing	—	—	Fluorescence
Ti64-CT	Ti6Al4V	Chemical Treatment (nanotexture)	—	—	Fluorescence, SEM, contact angle
Ti64-MP-C	Ti6Al4V	Mechanical Polishing	Mentha EO	—	Fluorescence, FTIR, Tape
Ti64-CT-C	Ti6Al4V	Chemical Treatment (nanotexture)	Mentha EO	—	Fluorescence, SEM, FTIR, contact angle, Tape
Ti64-MP-C-3 h/7 h/3 d/7 d	Ti6Al4V	Mechanical Polishing	Mentha EO	3 h/7 h/3 d/7 d water soaking	Fluorescence (7 d), FTIR, Tape
Ti64-CT-C-3 h/7 h /3 d/7 d	Ti6Al4V	Chemical Treatment (nanotexture)	Mentha EO	3 h/7 h/3 d/7 d water soaking	Fluorescence (7 d), FTIR, contact angle, tape
AISI316L-MP	AISI316LVM	Mechanical Polishing	—	—	Fluorescence
AISI316L-MG	AISI316LVM	Mechanical Grinding (SiC paper #400)	—	—	Fluorescence, SEM, scratch
AISI316L-MP-C	AISI316LVM	Mechanical Polishing	Mentha EO	—	Fluorescence, FTIR, Tape
AISI316L-MG-C	AISI316LVM	Mechanical Grinding (SiC paper #400)	Mentha EO	—	Fluorescence, SEM, Tape, scratch
AISI316L-MP-C-3 h/7 h/3 d/7 d	AISI316LVM	Mechanical Polishing	Mentha EO	3 h/7 h/3 d/7 d water soaking	Fluorescence (7 d), FTIR
AISI316L-MG-C-3 h/7 h/3 d/7 d	AISI316LVM	Mechanical Grinding (SiC paper #400)	Mentha EO	3 h/7 h/3 d/7 d water soaking	Fluorescence (7 d), Tape
AISI316L-MP-C-H_2_SO_4_/NaOH/deg	AISI316LVM	Mechanical Polishing	Mentha EO	H_2_SO_4_/NaOH/degreaser washing	FTIR, Tape
